# Lytic Activity of Polyvalent Staphylococcal Bacteriophage PhiSA012 and Its Endolysin Lys-PhiSA012 Against Antibiotic-Resistant Staphylococcal Clinical Isolates From Canine Skin Infection Sites

**DOI:** 10.3389/fmed.2020.00234

**Published:** 2020-06-10

**Authors:** Tomohiro Nakamura, Junya Kitana, Jumpei Fujiki, Masayuki Takase, Keita Iyori, Kenta Simoike, Hidetomo Iwano

**Affiliations:** ^1^Laboratory of Veterinary Biochemistry, School of Veterinary Medicine, Rakuno Gakuen University, Ebetsu, Japan; ^2^ELMS Animal Medical Center, Suginami City, Japan; ^3^Vet Derm Tokyo CO., Ltd., Fujisawa, Japan

**Keywords:** antibiotic-resistant bacteria, bacteriophage, endolysin, canine skin infection, *Staphylococcus pseudintermedius*

## Abstract

The spread of antibiotic-resistant bacteria (ARB) in human and veterinary medicine is of global concern. Notably, the emergence of methicillin-resistant *Staphylococcus pseudintermedius* has become a serious problem. In this context, bacteriophages and their lytic enzymes, endolysins, have received considerable attention as therapeutics for infectious diseases in place of antibiotics. The aim of the present study was to investigate the antibiotic-resistance patterns of staphylococcal species isolated from canine skin at a primary care animal hospital in Tokyo, Japan and evaluate the lytic activity of the staphylococcal bacteriophage phiSA012 and its endolysin Lys-phiSA012 against isolated antibiotic-resistant staphylococcal strains. Forty clinical staphylococcal samples were isolated from infection sites of dogs (20 from skin and 20 from the external ear canal). Susceptibility to antimicrobial agents was determined by a disk diffusion method. The host range of phiSA012 was determined by using a spot test against staphylococcal isolates. Against staphylococcal isolates that showed resistance toward five classes or more of antimicrobials, the lytic activity of phiSA012 and Lys-phiSA012 was evaluated using a turbidity reduction assay. Twenty-three *S. pseudintermedius*, 16 *Staphylococcus schleiferi*, and 1 *Staphylococcus intermedius* were detected from canine skin and ear infections, and results revealed 43.5% methicillin resistance in *S. pseudintermedius* and 31.3% in *S. schleiferi*. In addition, the prevalence multidrug resistance (MDR) *S. pseudintermedius* was 65.2%. PhiSA012 could infect all staphylococcal isolates by spot testing, but showed little lytic activity by turbidity reduction assay against MDR *S. pseudintermedius* isolates. On the other hand, Lys-phiSA012 showed lytic activity and reduced significantly the number of staphylococcal colony-forming units. These results demonstrated that ARB issues underlying in small animal hospital and proposed substitutes for antibiotics. Lys-phiSA012 has broader lytic activity than phiSA012 against staphylococcal isolates; therefore, Lys-phiSA012 is a more potential candidate therapeutic agent for several staphylococcal infections including that of canine skin.

## Introduction

Administration of systemic and/or topical antimicrobial agents is the most common therapeutic treatment for bacterial infectious diseases. Besides, antibiotics are often contained in feed for growth promotion of livestock (1). It has been previously reported that antibiotics are used in livestock more so than in humans (2–4). In this context, the overuse and misuse of antibiotics in humans and animals have resulted in the global wide-spread emergence of antibiotic-resistant bacteria (ARB) (5). A previous study by the UK government estimated that ARB would cause tens of millions of deaths per year and pose a greater human health risk than cancer by 2050 (6, 7).

*Staphylococcus aureus* is a well-known member of the human skin microbiota (8–10). However, methicillin-resistant *S. aureus* (MRSA) is one of the most frequently isolated ARB and can cause nosocomial infections (11). Meanwhile, *Staphylococcus pseudintermedius* is most frequently isolated as part of the normal canine skin flora and also as a pathogen of pyoderma, bacterial otitis, wounds, and abscess. As with MRSA in humans, methicillin-resistant *S. pseudintermedius* (MRSP) is frequently isolated from canine skin infections. It has been suggested that MRSP has acquired multi-drug resistance (MDR) to antimicrobials (12). Therefore, it is imperative that alternative therapeutic agents against staphylococci including MRSP are identified in the field of companion animal medicine.

At present, the use of bacteriophages as therapy for infectious diseases is receiving significant attention (13, 14). Bacteriophages are viruses that specifically infect bacteria and are lethal (15, 16). Previously, we reported the isolation of a bacteriophage against *S. aureus*, phiSA012, which showed efficient lytic activity toward various *S. aureus* strains *in vitro* (17) and demonstrated the therapeutic effect of phiSA012 on a mouse mastitis model caused by *S. aureus* (18).

Endolysins are bacteriophage-encoded and are translated at the end of the phage life cycle to lyse host bacteria, by hydrolyzing cell wall peptidoglycan from within or without, leading to the release of phage offspring (19–21). Most of the endolysins targeting Gram-positive bacteria harbor two functional domains: an enzymatically active domain (EAD), which hydrolyzes specific peptidoglycan bonds, and a cell wall–binding domain (CBD), which recognizes and binds specific peptidoglycan ligands or secondary cell wall polymers such as teichoic acids and determines the spectrum of endolysins within particular bacterial species or strains (22, 23). *Staphylococcus aureus* bacteriophage endolysins such as Lys-phiK, Lys-GH15, and Lys-phiSA012 harbor a multidomain composed of a cysteine, histidine-dependent amidohydrolase/peptidase domain and an amidase (AMID) domain as EADs, and an SH3b as a CBD at the C-terminus (21, 24, 25). It has been suggested that bacteria cannot easily acquire endolysin resistance (26, 27). In fact, it was reported that several bacteria could not develop resistance against endolysins after repeated exposure, as endolysins may cleave peptidoglycan sites that are essential for bacterial survival (23, 28, 29). At present, thus, endolysins are garnering abundant attention as a substitute for antibiotics in the treatment of bacterial infections (30–32). Previously, we reported that an endolysin, Lys-phiSA012, which was derived from phiSA012 and is categorized as a Lys-phiK like endolysin, exhibited high lytic activity against *S. aureus* including MRSA, a *S. pseudintermedius* strain, and a *Staphylococcus haemolyticus* strain (25).

The objective of the present study was to investigate the antibiotic-resistance patterns of staphylococcal species, isolated from canine skin at a primary care animal hospital, to confirm the spread of MRSP and MDR *S. pseudintermedius* in companion animals. In addition, we examined the utility of polyvalent phiSA012 and its endolysin Lys-phiSA012 as a treatment against clinical staphylococcal isolates, thereby expanding the available treatment options against ARB.

## Materials and Methods

### Sample Collection and Bacterial Identification

All samples were isolated from dogs visiting the ELMS Animal Medical Center, Tokyo, Japan. A total of 40 clinical isolates were collected aseptically from infected sites of dogs (20 from skin and 20 from the external ear canal) from April 2017 to August 2018. The animal study was reviewed and approved by Animal Care and Use Committee of Rakuno Gakuen University (Approval No. VH19B22). Each lesion site was sampled using a Culture Swab Plus (Nippon Becton Dickinson Company, Ltd., Tokyo, Japan) and sent to Vet Derm Tokyo, Ltd. for bacterial identification and determination of antibiotic resistance profile.

Clinical samples were cultivated on 5% sheep blood agar and incubated at 37°C for 24 h. Staphylococci were identified by BBLCRYSTAL (Nippon Becton Dickinson Company, Ltd.), and *nuc*-targeted multiplex polymerase chain reaction amplification was conducted for genotypic confirmation of *S. aureus, S. pseudintermedius*, and *Staphylococcus schleiferi* identification (33). *Staphylococcus pseudintermedius* used in the turbidity reduction assay was classified using the random amplification polymorphic DNA (RAPD) method to detect polymorphisms in *S. pseudintermedius* isolates (33).

### Antimicrobial Susceptibility Testing

Susceptibility to a panel of 21 antimicrobial agents was determined by a disk diffusion method according to the guidelines of the Clinical Laboratory Standards Institute (34, 35). The examined antibiotics were oxacillin (1 μg), amoxicillin/clavulanic acid (20/10 μg), cephalexin (30 μg), cefpodoxime (10 μg), cefovecin (30 μg), faropenem (5 μg), enrofloxacin (5 μg), orbifloxacin (10 μg), marbofloxacin (5 μg), gentamicin (10 μg), fradiomycin (30 μg), sulfamethoxazole/trimethoprim (23.75/1.25 μg), clindamycin (2 μg), lincomycin (2 μg), erythromycin (15 μg), doxycycline (30 μg), minocycline (30 μg), chloramphenicol (30 μg), florfenicol (30 μg), fosfomycin (50 μg), and rifampicin (5 μg).

### Bacteriophage and Endolysin Preparation

The *S. aureus* virulent phage phiSA012 (accession number NC_023573.1) was isolated from sewage in Tokyo, Japan in a previous study (17). PhiSA012 was propagated by the plate lysate method (36). In brief, 100 μL of phage lysate was mixed with 100 μL of an overnight culture of *S. aureus* strain SA003 (17) in 3 mL of 0.5% top agar, then plated on Luria-Bertani (LB) agar, and incubated at 37°C overnight. After 3 mL of salt magnesium buffer [100 mM NaCl, 8 mM MgSO_4_, 50 mM Tris-HCl (pH 7.5), 0.01% gelatin] was added to the plate, the overlayer was scraped off to extract the phage, and the supernatant was collected by centrifugation (10,000 × g for 5 min at 4°C). The supernatant was purified by CsCl density gradient centrifugation (36). Purified phiSA012 was titrated and stored at 4°C until use.

Lys-phiSA012 was expressed and purified as described in our previous report (25). In brief, *Escherichia coli* BL21(DE3) strain, which possesses the Lys-phiSA012 encoding plasmid, was cultured in LB medium containing 100 μg/mL of ampicillin, and protein expression was induced by the addition of isopropyl β-thiogalactopyranoside to a final concentration of 0.1 mM at the logarithmic phase (corresponding to 0.4–0.6 OD_600_) and then incubated overnight at 25°C with shaking. Cells were harvested by centrifugation (2,300 × g for 5 min at 4°C) and the pellets were lysed via sonication. The lysate was centrifuged at 16,000 × g for 30 min at 4°C, and then the supernatant containing soluble GST-tagged protein was collected using a chromatographic column packed with Glutathione Sepharose 4B. Pure Lys-phiSA012 protein was obtained by loading the PreScission Protease mix [80 μL (160 units)] of PreScission Protease and 920 μL of cleavage buffer (50 mM Tris-HCl, 150 mM NaCl, 1 mM dithiothreitol) onto the column and then stored at −30°C until use.

### Spot Testing

Four microliters of phiSA012 suspension at a titer of 10^9^ plaque-forming unit (PFU)/mL was dropped onto a double-layer LB agar plate containing the staphylococcal isolates and *S. aureus* SA003 as a positive control. After overnight incubation at 37°C, the infected area was characterized as one of four categories: clear plaque (C), turbid plaque (T), faint plaque (F), or no plaque (N).

### Turbidity Reduction Assay

All staphylococcal isolates that showed resistance against five classes or more of antimicrobials (SS3014, SP3018, SP3401, SP3567, SP4531, SP5158, SP5405, SP5432, SP5515, SP7369, SP7454, SP7542, and SP7971) and SA003 were grown in LB medium. At a concentration of approximately 10^8^ colony-forming units (CFU/mL), each bacterium was mixed with phiSA012 suspension at a final titer of 10^8^ PFU/ml (Multiplicity of infection ≒ 1.0), and then the OD_595_ value was monitored using a plate reader (Sunrise Rainbow Thermos RC; TECAN Austria GmbH, Salzburg, Austria) with incubation.

The lytic activity of Lys-phiSA012 was assessed using a turbidity reduction assay as described previously (25). In brief, the same as above staphylococcal isolates were grown in LB medium at 37°C to an OD_600_ of 1.0. Each culture was centrifuged at 2,300 × g for 5 min at 4°C, and the cells were resuspended in 2 × LB medium, and then stored on ice until use. The turbidity reduction assay was initiated by adding the same amount of purified Lys-phiSA012 (100 μg/mL), and then the OD_595_ value was monitored using a plate reader with incubation. The decrease in viable cells corresponding to the loss of turbidity was tested by plating the aliquots from the reaction solution of turbidity assay at the end of monitoring (2 h) for counting of CFUs.

### Statistical Analysis

Statistical analysis was performed using two-tailed Student *t*-tests or Welch *t*-tests according to the result of *F*-tests, and Dunnett test from three independent experiments. *p* < 0.05 was considered to be statistically significant.

## Results

### Strain Identification and Antimicrobial Susceptibility Testing

**Figure 1:**
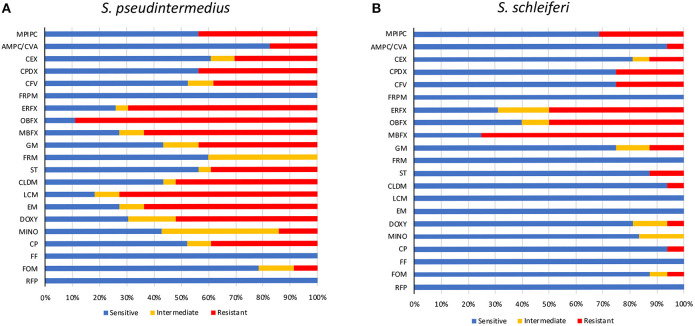
Antimicrobial susceptibility of staphylococcal isolates. Antimicrobial susceptibility of *S. pseudintermedius* **(A)** and *S. schleiferi* **(B)**. MPIPC, oxacillin; AMPC/CVA, amoxycillin/clavulanic acid; CEX, cephalexin; CPDX, cefpodoxime; CFV, cefovecin; FRPM, faropenem; ERFX, enrofloxacin; OBFX, orbifloxacin; MBFX, marbofloxacin; GM, gentamicin; FRM, fradiomycin; ST, sulfamethoxazole/trimethoprim; CLDM, clindamycin; LCM, lincomycin; EM, erythromycin; DOXY, doxycycline; MINO, minocycline; CP, chloramphenicol; FF, florfenicol; FOM, fosfomycin; RFP, rifampicin.

The prevalence of MRSP in veterinary teaching hospitals has been confirmed in several reports, but that in primary care animal hospitals remains unclear. Therefore, we investigated the antibiotic-resistance patterns of staphylococcal isolates at a primary care animal hospital in Tokyo, Japan. The most frequently isolated *Staphylococcus* species was *S. pseudintermedius* (23/40), followed by *S. schleiferi* (16/40), and *Staphylococcus intermedius* (1/40). The antibiotic-resistance pattern is summarized in Table 1 and Figure 1. Methicillin resistance as determined by oxacillin disk susceptibility was 43.5% in *S. pseudintermedius* (10/23) and 31.3% in *S. schleiferi* (5/16). Surprisingly, both *S. pseudintermedius* and *S. schleiferi* exhibited obvious resistance to fluoroquinolones (enrofloxacin, orbifloxacin, and marbofloxacin). Next, we attempted to determine whether these isolates demonstrated MDR based on the definition proposed by the European Center for Disease Prevention and Control and the Centers for Disease Control and Prevention (37). The prevalence of MDR was 65.2% (15/23) for *S. pseudintermedius* and 12.5% (2/16) for *S. schleiferi* (Supplementary Table S1). On the other hand, no resistance was observed toward faropenem, florfenicol, and rifampicin for all staphylococcal isolates.

**Table 1:** Antimicrobial susceptibility of staphylococcal isolates.

	**Number of isolates (%)**											
	***S. pseudintermedius***				***S. schleiferi***				***S. intermedius***			
**Antimicrobials**	**S**	**I**	**R**	**Total**	**S**	**I**	**R**	**Total**	**S**	**I**	**R**	**Total**
MPIPC	13	0	10 (43.5%)	23	11	0	5 (31.3%)	16	0	0	1 (100%)	1
AMPC/CVA	19	0	4 (17.4%)	23	15	0	1 (6.3%)	16	0	0	1 (100%)	1
CEX	14	2	7 (30.4%)	23	13	1	2 (12.5%)	16	0	0	1 (100%)	1
CPDX	13	0	10 (43.5%)	23	12	0	4 (25%)	16	0	0	1 (100%)	1
CFV	11	2	8 (38.1%)	21	9	0	3 (25%)	12	0	0	1 (100%)	1
FRPM	21	0	0 (0%)	21	12	0	0 (0%)	12	1	0	0 (0%)	1
ERFX	6	1	16 (69.6%)	23	5	3	8 (50%)	16	0	0	1 (100%)	1
OBFX	1	0	8 (88.9%)	9	4	1	5 (50%)	10	0	0	0	0
MBFX	3	1	7 (63.6%)	11	1	0	3 (75%)	4	0	0	1 (100%)	1
GM	10	3	10 (43.5%)	23	12	2	2 (12.5%)	16	0	0	1 (100%)	1
FRM	6	4	0 (0%)	10	8	0	0 (0%)	8	0	0	0	0
ST	13	1	9 (39.1%)	23	14	0	2 (12.5%)	16	1	0	0 (0%)	1
CLDM	10	1	12 (52.2%)	23	15	0	1 (6.3%)	16	0	0	1 (100%)	1
LCM	2	1	8 (72.7%)	11	4	0	0 (0%)	4	0	0	1 (100%)	1
EM	3	1	7 (63.6%)	11	4	0	0 (0%)	4	0	0	1 (100%)	1
DOXY	7	4	12 (52.2%)	23	13	2	1 (6.3%)	16	0	0	1 (100%)	1
MINO	6	6	2 (14.3%)	14	5	1	0 (0%)	6	0	1	0 (0%)	1
CP	12	2	9 (39.1 %)	23	15	0	1 (6.3%)	16	0	0	1 (100%)	1
FF	10	0	0 (0%)	10	8	0	0 (0%)	8	0	0	0	0
FOM	18	3	2 (8.7%)	23	14	1	1 (6.3%)	16	1	0	0 (0%)	1
RFP	21	0	0 (0%)	21	12	0	0 (0%)	12	1	0	0	1

*MPIPC, oxacillin; AMPC/CVA, amoxycillin/clavulanic acid; CEX, cephalexin; CPDX, cefpodoxime; CFV, cefovecin; FRPM, faropenem; ERFX, enrofloxacin; OBFX, orbifloxacin; MBFX, marbofloxacin; GM, gentamicin; FRM, fradiomycin; ST, sulfamethoxazole/trimethoprim; CLDM, clindamycin; LCM, lincomycin; EM, erythromycin; DOXY, doxycycline; MINO, minocycline; CP, chloramphenicol; FF, florfenicol; FOM, fosfomycin; RFP, rifampicin*.

### Host Range and Lytic Activity of the Bacteriophage phiSA012 Against Staphylococcal Isolates

**Figure 2:**
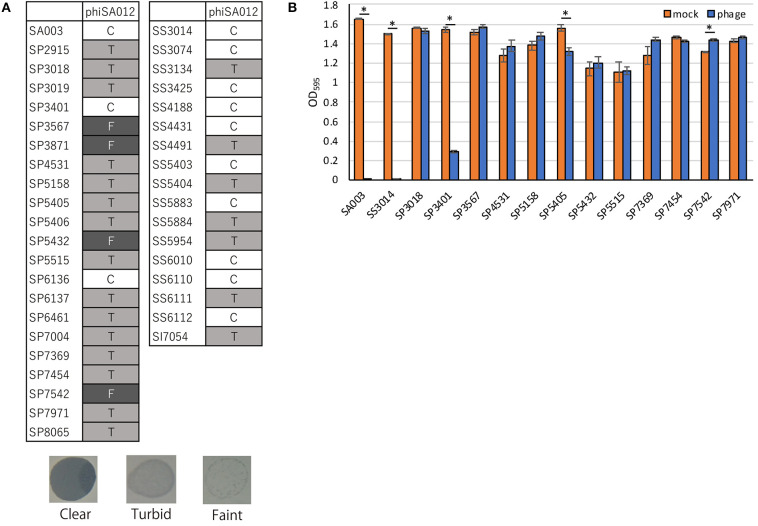
Host range and lytic activity of phiSA012 against staphylococcal isolates from canine skin infection sites. Host range and lytic activity of phiSA012 were evaluated by spot testing **(A)** and turbidity reduction assay **(B)**. **(A)** Clear plaques “C” indicates the highest lysis activity, followed by turbid plaques “T” and faint plaques “F” in spot testing. Representative images of formed spots are showed below. **(B)** The graph shows OD_595_ value of mock treated or phage treated bacterial cultures at the end of monitoring (24 h). The columns indicate the mean and error bars indicate standard error (SE). Statistical significance (*p < 0.01) is indicated with an asterisk by Student's *t*-test.

PhiSA012 has been reported as a broad-range lytic phage against *S. aureus* strains (17, 18). In this study, we evaluated the host range of phiSA012 against all staphylococcal isolates except two *S. pseudintermedius* strains (SP3399 and SP6931), which could not be re-cultured. PhiSA012 could infect all staphylococcal isolates (*S. pseudintermedius, S. schleiferi*, and *S. intermedius*) including methicillin-resistant and MDR staphylococci (Figure 2A). Against *S. pseudintermedius*, clear plaques, turbid plaques and faint plaques accounted for 9.5% (2/21), 71.4% (15/21), and 19.0% (4/21), respectively.

In addition to spot-testing evaluation, turbidity reduction assays were performed to observe sequential and quantitative lytic activity of phiSA012 (Supplementary Figure S1). As target bacteria, 12 MDR *S. pseudintermediu*s strains and one MDR *S. schleiferi* that showed resistance against five classes or more of antimicrobials were selected, and SA003 was used as a positive control. Twelve *S. pseudintermedius* strains exhibited different polymorphisms in the RAPD method (Supplementary Figures S2A,B). PhiSA012 significantly inhibited bacterial growth of SA003, SS3014, SP3401, and SP5405 compared with mock (Figure 2B). On the other hand, phiSA012 showed slightly bacterial growth inhibition in a few hours after phage infection against others isolates, but there was little difference in OD_595_ values compared with mock at the 24 h point (Figure 2B, Supplementary Figure S1). These results demonstrated that phiSA012 could infect all staphylococcal isolates, but showed little lytic activity against most of the *S. pseudintermedius* isolates.

### Lytic Activity of the Endolysin Lys-phiSA012 Against MDR *S. pseudintermedius*

**Figure 3:**
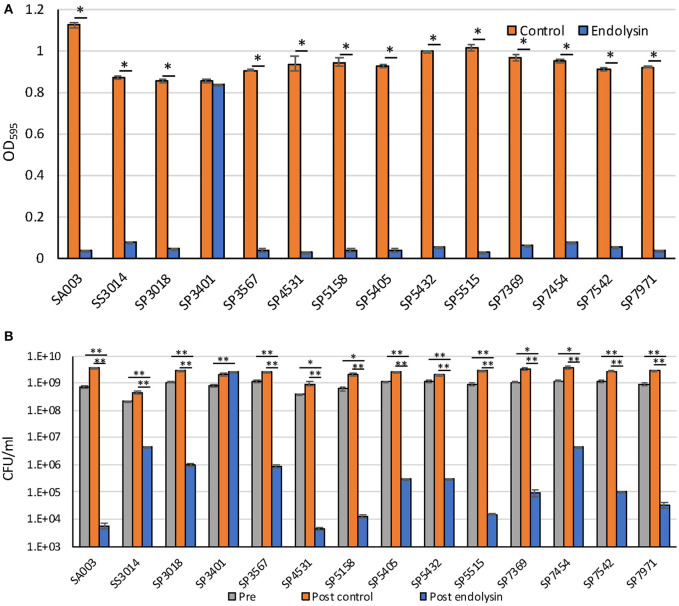
Lytic activity of Lys-phiSA012 against MDR staphylococcal isolates. Lytic activity of Lys-phiSA012 was evaluated by turbidity reduction assay **(A)** and following CFUs counting **(B)**. The columns indicate the mean and error bars indicate standard error (SE). **(A)** The graph shows OD_595_ value of buffer control treated or endolysin treated bacterial cultures at the end of monitoring (2 h). Statistical significance (*p < 0.01) is indicated with an asterisk by Student's *t*-test or Welch's *t*-test. **(B)** The graph shows CFUs of pretreated, post–buffer control treated or post–endolysin-treated bacterial cultures at the end of monitoring (2 h). Statistical significance (*p < 0.05 and **p < 0.01) is indicated with an asterisk by Dunnett's test.

Previously, we reported that Lys-phiSA012 showed lytic activity against *S. pseudintermedius* (25). In this study, we examined whether Lys-phiSA012 showed lytic activity against 13 MDR staphylococcal clinical isolates. Except SP3401, Lys-phiSA012 showed rapid antimicrobial activity in turbidity reduction assay against all MDR *S. pseudintermedius* and *S. schleiferi* within 2 h (Figure 3A, Supplementary Figure S3). Next, we confirmed the decrease in viable cells corresponding to the loss of turbidity by counting CFUs at the end of turbidity monitoring (2 h). Lys-phiSA012 significantly yielded 2–5.5 log reduction of CFUs compared to buffer controls, except SP3401 (Figure 3B).

## Discussion

At present, the emergence of ARB is one of the most pressing global health issues. Particularly, ESKAPE pathogens (*Enterococcus faecium, S. aureus, Klebsiella pneumoniae, Acinetobacter baumannii, Pseudomonas aeruginosa*, and *Enterobacter* species) have the potential to develop several drug resistance mechanisms and cause lethal nosocomial infections (38). We have demonstrated the therapeutic potential of bacteriophages and endolysins against *P. aeruginosa* and *S. aureus* as alternatives to antimicrobials (17, 18, 25, 36, 39). In the present study, we focused on antibiotic-resistant staphylococci isolated from canine skin infectious diseases and evaluated the lytic activity of the bacteriophage phiSA012 and its endolysin Lys-phiSA012.

The most frequently isolated strains in this study were *S. pseudintermedius*, and the second were *S. schleiferi*. *Staphylococcus pseudintermedius* is well-known as the main cause of canine pyoderma and external otitis, and *S. schleiferi* is also a causative agent of these infections. As with MRSA in human medicine, the emergence of MRSP is of concern in veterinary medicine. According to several reports, the MRSP isolation frequencies were 10.4% in Spain, 17.6% in Korea, 27.3–38.2% in the United States, 47.9% in South China, and 57% in Japan (12, 40–43). In this study, the frequency of MRSP was 43.5%, which was lower than that in teaching hospitals in Japan (57%). In university or teaching hospitals, many cases are treated with antibiotics prior to referral, and this may result in a high prevalence of MRSP compared with primary care hospitals. Several reports described that *S. schleiferi* has a high rate of methicillin resistance compared with *S. pseudintermedius* (44, 45); however, in this investigation, the rate of MRSP was higher than methicillin-resistant *S. schleiferi* (Figure 1). In addition, high rates of fluoroquinolone-resistant *S. pseudintermedius* and *S. schleiferi* were detected in this investigation. Moreover, and most remarkably, the rate of MDR *S. pseudintermedius* was 65.2%, which was greater than the rate of MRSP. According to the present study, the emergence of antibiotic-resistant staphylococci is becoming a serious problem in primary care animal hospitals, necessitating confirmation of the need for antibiotics in the treatment of individual cases as well as ongoing surveillance of ARB prevalence.

Adsorption of a bacteriophage to the host cell is the first step of infection and is one of the important processes that determines the host range (46). Staphylococci have various glycoepitopes (GlcNAc, GalNAc, or Glc) and two types of wall teichoic acid (WTA) backbone [ribitol–phosphate (RboP) and glycerol–phosphate (GroP)] (47, 48). The infectivity of staphylococcal *Siphoviridae* and *Podoviridae* phages depends on the WTA type of the host cell; for example *Siphoviridae* phage Φ11 only recognizes GlcNAc of RboP WTA (49). On the other hand, phiSA012, which is classified as a *Myoviridae* phage, utilizes the back bone of WTA as a receptor and can infect several staphylococci regardless of the WTA type (46, 50). We and another research group reported that phiSA012 has a broad host range against various *S. aureus* strains including MRSA (17, 18). In addition to the previous reports, we found that phiSA012 could infect MDR *S. pseudintermedius, S. schleiferi*, and *S. intermedius* by spot testing. To apply bacteriophages as therapeutics for canine pyoderma or otitis externa, it is important determine not only the host range toward staphylococci but also the strength of the lytic activity, especially against *S. pseudintermedius* and *S. schleiferi*. However, the lytic activity of phiSA012 against *S. pseudintermedius* might be somewhat weak, as the rate of making clear plaques during spot testing was fewer than against *S. aureus* in the present study and previous reports (18, 50). In addition, turbidity reduction assay confirmed that phiSA012 has little activity against most of the staphylococcal isolates which were showed turbid or faint plaques by spot testing (Figure 2, Supplementary Figure S1). On the other hand, *S. pseudintermedius* phages ΦSP0120, ΦSP0197, and ΦSP276 could infect only *S. pseudintermedius* of the staphylococci; however, they showed clear plaques toward almost all *S. pseudintermedius* strains (50). Therefore, it might be preferable to find and use bacteriophages that show high lytic activity toward *S. pseudintermedius* to treat canine pyoderma or externa otitis.

We previously demonstrated that Lys-phiSA012 had lytic activity against *S. aureus, S. pseudintermedius*, and *S. haemolyticus* (25). In this study, we examined whether Lys-phiSA012 has lytic activity against several MDR staphylococcal clinical strains isolated from canine skin. We observed that Lys-phiSA012 showed lytic activity and reduced significantly the number of CFUs compared to buffer control against almost all MDR staphylococcal isolates (Figure 3, Supplementary Figure S3). In addition, phiSA012 showed little lytic activity against almost of the *S. pseudintermedius* isolates, whereas Lys-phiSA012 showed clearly lytic activity toward staphylococcal isolates including these strains. Lys-phiSA012 harbors the SH3b domain as a CBD, and this domain has been shown to recognize and bind the pentaglycine bridge of peptidoglycan, a characteristic structure of most staphylococci (51). That is to say, Lys-phiSA012 probably shows lytic activity against almost all staphylococci that have the pentaglycine bridge in peptidoglycan and a broader host range than phiSA012. On the other hands, it has been previously reported that WTAs conformations affect binding of endolysins to the cells and prevents lysis from without by endolysins (52, 53). In this study, only SP3401 could not be lysed by Lys-phiSA012 but lysed by phiSA012, suggesting that WTAs on SP3401 play a role in phiSA012 infection as a receptor while might inhibit the lysis from without mediated by the Lys-phiSA012. Further investigations are required to elucidate the mechanisms underlying lysis from without, which would contribute to appropriate use of phiSA012 and Lys-phiSA012.

## Conclusion

Our investigation indicated that the emergence of MDR *S. pseudintermedius* is becoming a serious problem in primary care animal hospitals as observed for MRSP. In the context of ARB emergence, we propose that phage therapy has therapeutic potential as an alternative to antibiotics. We confirmed that phiSA012 has a broad range toward staphylococci; however, further studies are warranted to verify whether its lytic activity is sufficient to treat canine skin staphylococcal infections. Moreover, Lys-phiSA012 showed lytic activity against most of the MDR *S. pseudintermedius* and *S. schleiferi* strains. This finding supports the use of Lys-phiSA012 as a candidate therapeutic agent for canine skin staphylococcal infections.

## Data Availability Statement

All datasets generated for this study are included in the article/Supplementary Material.

## Author Contributions

TN, JF, and HI designed experiments. TN and MT collected skin swab samples. KI and KS performed bacterial identification and antimicrobial susceptibility testing. TN and JK performed experiments and analyzed the data. TN and HI wrote the paper.

## Conflict of Interest

Authors KI and KS were employed by company Vet Derm Tokyo, Ltd. The remaining authors declare that the research was conducted in the absence of any commercial or financial relationships that could be construed as a potential conflict of interest.
