# Supplementary material for: Lytic Activity of Polyvalent Staphylococcal Bacteriophage PhiSA012 and Its Endolysin Lys-PhiSA012 Against Antibiotic-Resistant Staphylococcal Clinical Isolates From Canine Skin Infection Sites
Source: Front Med (Lausanne). 2020 Jun 10;7:234. doi: 10.3389/fmed.2020.00234 (PMC7298730; doi:10.3389/fmed.2020.00234)
Supplement: Supplementary file 2 [file Data_Sheet_2.PDF]

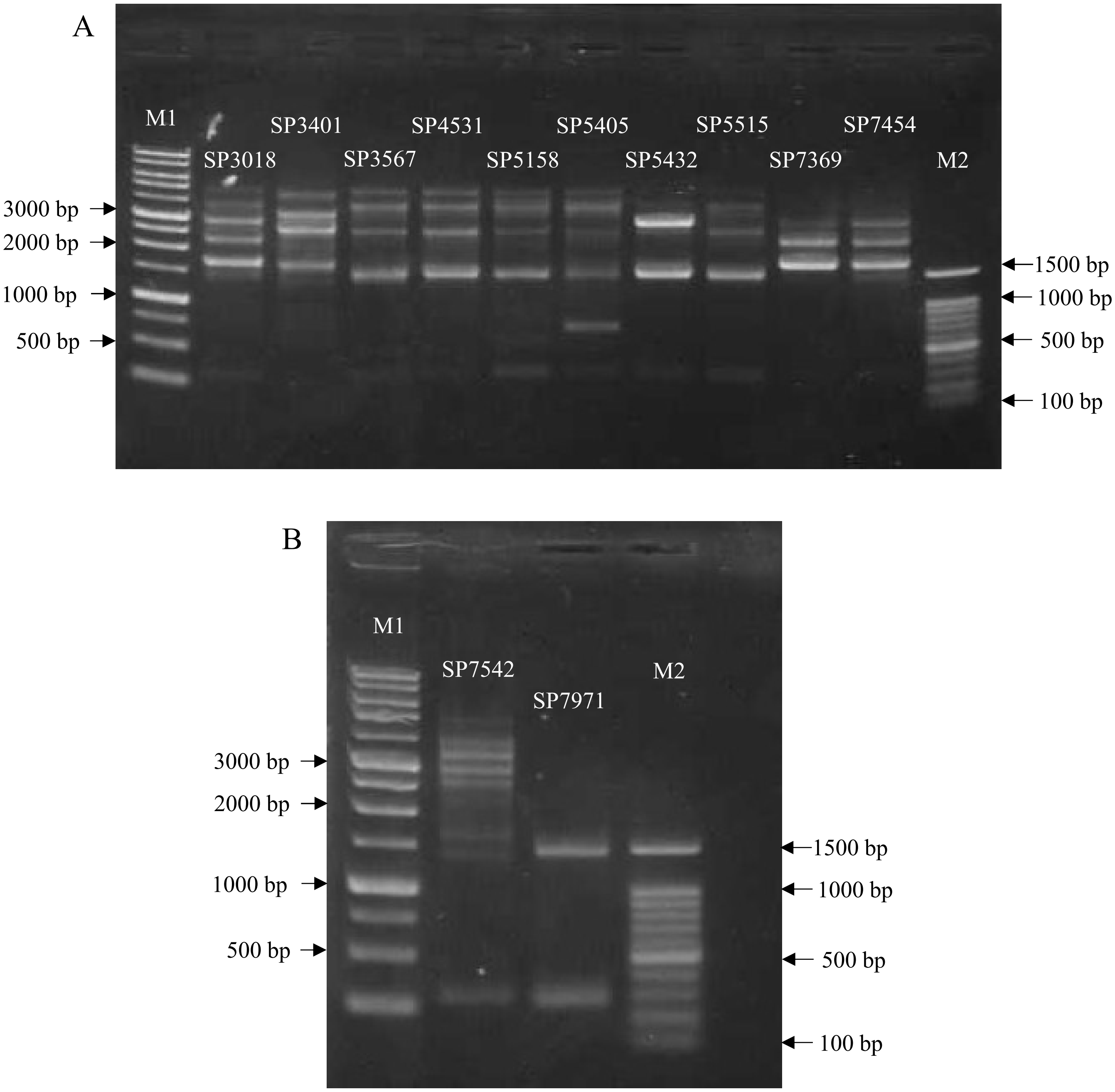

Supplementary Figure S2. Twelve MDR *S. pseudintermedius* strains were classified by random amplification polymorphic DNA (RAPD) method. The results showed that these strains have polymorphisms. DNA ladders (M1, 1-kb ladder; M2, 100-kb ladder) was used for molecular standard.
