# Supplementary material for: Lytic Activity of Polyvalent Staphylococcal Bacteriophage PhiSA012 and Its Endolysin Lys-PhiSA012 Against Antibiotic-Resistant Staphylococcal Clinical Isolates From Canine Skin Infection Sites
Source: Front Med (Lausanne). 2020 Jun 10;7:234. doi: 10.3389/fmed.2020.00234 (PMC7298730; doi:10.3389/fmed.2020.00234)
Supplement: Supplementary file 3 [file Data_Sheet_3.PDF]

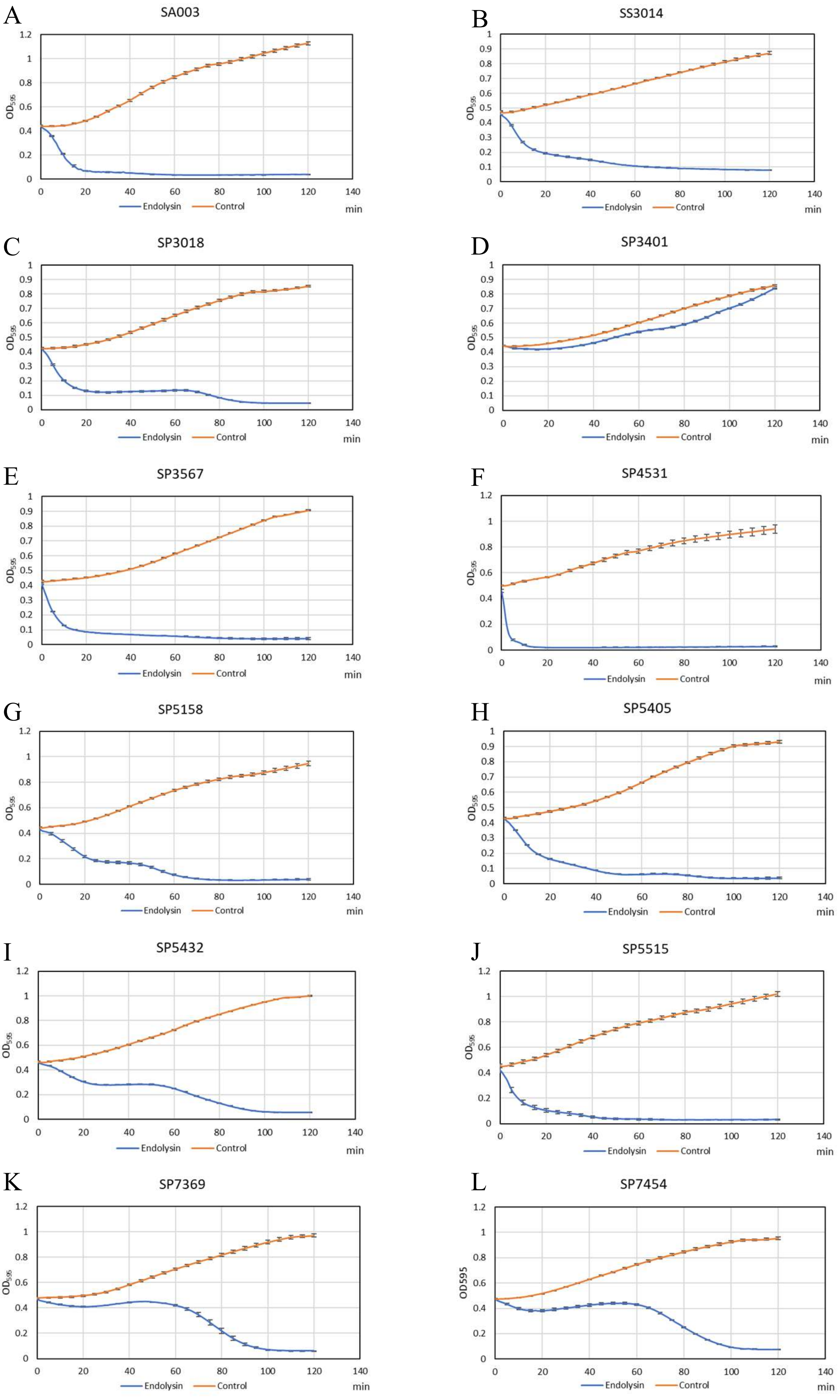
A
SA003
OD595
Endolysin
Control
min
B
SS3014
C
SP3018
D
SP3401
E
SP3567
F
SP4531
G
SP5158
H
SP5405
I
SP5432
J
SP5515
K
SP7369
L
SP7454
0
20
40
60
80
100
120
140
0.2
0.4
0.6
0.8
1
1.2

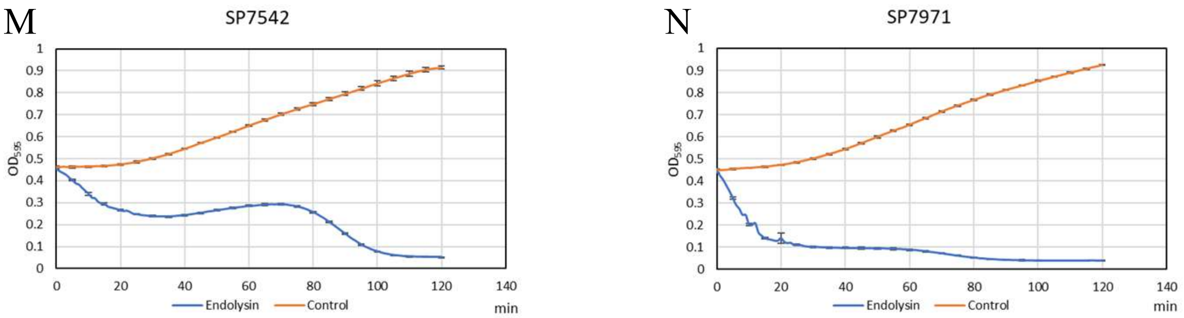

Supplementary Figure S3. Lytic activity of Lys-phiSA012 against MDR staphylococcal isolates. OD_{595} values were monitored following the addition of buffer control (Control) or phiSA012 (Endolysin) for 24 hours. The error bars indicate standard error (SE).
